# Radiation therapy response prediction for head and neck cancer using multimodal imaging and multiview dynamic graph autoencoder feature selection

**DOI:** 10.1002/mp.70026

**Published:** 2025-09-22

**Authors:** Amir Moslemi, Laurentius Oscar Osapoetra, Aryan Safakish, Lakshmanan Sannachi, David Alberico, Gregory J Czarnota

**Affiliations:** ^1^ Physical Sciences Sunnybrook Research Institute Sunnybrook Health Sciences Centre Toronto Canada; ^2^ Department of Radiation Oncology Sunnybrook Health Sciences Centre Toronto Canada; ^3^ Department of Physics Toronto Metropolitan University Toronto Canada; ^4^ Department of Medical Biophysics University of Toronto Toronto Canada

**Keywords:** autoencoder, CT, head and neck cancers, MRI, multiview feature selection, quantitative ultrasound spectroscopy, radiation therapy, radiomic features

## Abstract

**Background:**

External beam radiation therapy is a common treatment for head and neck (H&N) cancers. Radiomic features derived from biomedical images have shown promise as effective biomarkers used to assess tumor heterogeneity and predict response to treatment. However, most studies employ only a single biomedical imaging modality to determine radiomic features or naively concatenate radiomic features from different imaging modalities.

**Purpose:**

The objective of this study is to assess the effectiveness of multiview feature selection (MVFS) in identifying the most discriminative radiomic features determined from pretreatment quantitative ultrasound spectroscopic (QUS) parametric maps, as well as computed tomography (CT), and magnetic resonance imaging (MRI) modalities. These features were used to train predictive models to predict outcomes of radiation therapy for head and neck (H&N) cancer.

**Method:**

70, 70, and 350 radiomics features were extracted from pre‐treatment CT and MRI images, as well as seven QUS parametric maps, respectively. We proposed an MVFS technique named Adaptive Graph Autoencoder Multi‐View Feature Selection (AGAMVFS), based on dynamic graph learning and autoencoder. In AGAMVFS, adaptive and collaborative graphs are learned at multiple levels to discriminate among view‐specific features. An autoencoder is then applied to concatenated features to select the most discriminative ones. This approach fosters collaboration across different views and achieves a consensus projection for feature selection. Leave‐one‐patient‐out cross‐validation was applied to split the data into train and test sets and selected features were used to train two classifiers (support vector machine (SVM) and k‐nearest neighbor (KNN)) to build a predictive model, tasked with predicting response to treatment for patients with H&N cancers. Fivefold cross‐validation was applied on training set to tune the hyperparameters of SVM and KNN classifiers. Consequently, the performance of classifiers was evaluated using accuracy, F1‐score, balanced accuracy, sensitivity, and specificity metrics. Additionally, a two‐sided *t*‐test was applied to the selected features. We compared the proposed method with a single imaging modality and state‐of‐the‐art feature selection techniques.

**Results:**

We recruited 63 (59 male (94%) and 4 female (6%)) H&N cancer patients with bulky metastatic neck lymph node (LN) involvement. The mean age was 58.9 ± 10.2 years. The AGAMVFS with the SVM classifier obtained the best performance and achieved 76% sensitivity, 91% specificity, 85% accuracy, and 83% balanced accuracy. Results showed the effectiveness of proposed method with superiority over other feature selection techniques. The most top‐10 frequent features were six QUS radiomics, three MRI radiomics, and one CT radiomics features.

**Conclusion:**

The results demonstrated that the proposed predictive model is able to predict H&N cancer treatment response. MVFS provided better interpretabilityfor analysing features and preserved the inter‐correlation among features from different imaging modalities.

## 1 INTRODUCTION

The World Health Organization reported 930 000 new head and neck (H&N) cancer cases in 2020.^1^ H&N cancer is ranked as the sixth most common type of cancer.^2^ H&N cancers encompass cancers of the oral cavity, pharynx, larynx, paranasal sinuses, nasal cavity, and salivary glands.^3^ Squamous cell carcinoma is the predominant type of H&N cancer.^4^ Although distant metastasis is rarely found in H&N cancers at the time of diagnosis, regional LN involvement is frequently observed.^2^ Treatment for H&N cancers typically involves a combination of surgery, radiation therapy (RT), and systemic therapy. Factors such as viral infections,^5^ tobacco use, and alcohol consumption^6^ increase the risk of developing H&N cancers. RT efficacy is influenced by several factors, including cancer subtype, anatomical stage, and apparent risk factors.^7^ Clinicians utilize imaging for both diagnostic and treatment purposes. For treatment, imaging is used to identify and delineate the tumor,^8^, ^9^ before computing complex treatment plans with dosimetric calculations.^10^ For diagnosis, computed tomography (CT),^11^ magnetic resonance imaging (MRI),^12^ positron emission tomography (PET),^13^ and ultrasound (US)^14^ are clinically available modalities to best inform clinicians regarding the nature of disease.

In recent years, prediction of treatment outcomes using imaging biomarkers and expert system models has garnered considerable attention. Imaging biomarkers are useful for extracting textural information about tumor heterogeneity. Additionally, evidence shows that increased tumor heterogeneity stems from variability in both tumor microvasculature and genomic heterogeneity which contribute to a reduction in the probability of a successful response to treatment.^15^ Tumor heterogeneity leads to an increase in resistance of tumor against RT, decreasing the effectiveness of treatment.^16^ In this context, Safakish et al.^17^ extracted deep textural radiomics features from CT to predict the response to RT in H&N cancer patients using machine learning classifiers. The best features at each step were selected based on classifier performance, and the next set of radiomics features was subsequently extracted. Sellami et al.^18^ determined radiomics features from cone‐beam computed tomography (CBCT) to predict treatment response in H&N cancer patients. A total of 88 features were extracted from the gross tumor volume (GTV), and features with an area under the curve (AUC) greater than 0.65 were selected. Zahid et al.^19^ proposed a prediction system to predict RT treatment outcomes for H&N cancer patients using learned tumor growth rate and capacity reduction fraction extracted from CBCT. A mathematical approach was used to model tumor‐volume dynamics in response to treatment, in which an instantaneous function of therapy was defined to represent the dynamic carrying capacity. Zhang et al.^20^ proposed a prediction model to predict LN response to chemotherapy in patients with locally advanced H&N cancer using CT‐based radiomics. They trained a multivariable logistic regression using 93 CT radiomics features. Jalalifar et al.^21^ proposed a deep learning architecture to predict the outcome of stereotactic RT in patients with metastasis using MRI. They employed the InceptionResNetV2 network to extract features from each MRI slice and incorporated a transformer network to preserve the spatial dependencies among MRI slices. They trained a deep learning model on 99 patients and tested on 25 patients. In another study, Jalalifar et al.^22^ proposed an explainable deep learning technique based on a novel self‐attention‐guided 3D residual network to predict local failure in brain metastasis after RT using MRI. They considered convolutional block attention module and self attention to enhance the effectiveness of 3D residual network in processing multi‐channel MRI data. Additionally, they applied the concept of transferring pretrained models to the target task to improve model performance.

A major concern in the aforementioned studies is the reliance on single imaging modalities. In all these studies, features were extracted from a single imaging modality, and models were trained to predict RT treatment outcomes. However, using multiple imaging modalities can provide a more comprehensive feature set, enabling machine learning to be trained with more informative features. In this regard, Martens et al.^23^ extracted features from MRI and 18F‐FDG‐PET/CT before and 10 days after treatment to predict RT outcomes in H&N cancer patients. Imaging biomarkers included intravoxel incoherent motion, apparent diffusion coefficient, quantitative pharmacokinetic model, and whole‐lesion metabolic tumor volume. Wang et al.^24^ proposed a multi‐objective radiomics model to predict locoregional recurrence in H&N cancer patients using PET and CT. They utilized multiple classifiers to build a meta‐classifier by fusing all classifiers. A total of 257 features were extracted for each imaging modality and then concatenated to build the feature set. Finally, they applied the minimal‐redundancy‐maximal‐relevance (mRMR) feature selection technique to obtain the most discriminative features. Wong et al.^25^ assessed the effectiveness of multimodal imaging in predicting treatment response in patients with locally advanced H&N cancer. They recruited 35 patients and extracted imaging features from various imaging modalities, including 18F‐FDG‐PET/CT, diffusion‐weighted, dynamic contrast‐enhanced, and susceptibility‐weighted MRI. Similarly, they concatenated all features and built a predictive model based on the selected features. Trada et al.^26^ combined diffusion‐weighted imaging MRI and FDG‐PET/CT features to predict RT response in H&N cancer patients. They showed that a combination of changes in week 3 DWI and FDG‐PET/CT imaging parameters could be used to inform future risk‐adapted clinical trials in H&N cancer. Salmanpour et al.^27^ determined radiomic features from PET and CT fused images for survival prediction in H&N cancer. They applied an image‐level fusion technique to fuse PET and CT, extracting 215 radiomics features from the fused PET–CT. Additionally, they used an autoencoder to extract deep features and reduce the feature dimensions. In another study, Salmanpour et al.^28^ extracted radiomics features from PET only, CT only, and fused PET–CT to predict survival in H&N cancer patients. They concatenated all radiomics features and applied the intraclass correlation coefficient to remove highly correlated features and analysis of variance to select the most discriminative features.

In all the above multimodal imaging studies, features from different modalities were naïvely concatenated, and then feature selection techniques were applied. This simple concatenation fails to preserve and identify the correlations within different imaging modalities. Consequently, the classifier's ability to predict treatment response is notably compromised when it overlooks the correlation among various features from different imaging modalities. Differences among imaging modalities are neglected by concatenating features from different imaging modalities and the associations between different modalities are subsequently discarded. Therefore, concatenation of features from different imaging modalities failed in the joint selection of heterogeneous features.

To address these issues, for the first time we conducted a study to predict RT treatment response in H&N cancer patients using radiomics features determined from pretreatment QUS parametric maps, CT, and MRI images with a novel multiview feature selection (MVFS). We determined MRI, CT, and QUS radiomics features to create a comprehensive feature set and proposed a novel MVFS based on an autoencoder with dynamic graph learning and multilevel projections. The proposed MVFS is a robust method to select the most discriminative features across all modalities while considering the correlation among different imaging modalities. Additionally, it employs modality‐collaborative radiomics to explore the consensus embedding space of data, with the goal of learning view‐specific projections.

The objective of this study is to assess the effect of multimodal imaging and MVFS on predicting RT response in H&N cancer patients. We hypothesize that the efficiency of the predictive model will improve in predicting RT response in H&N cancer patients through the use of multimodal imaging and MVFS.

The contributions of our study are listed as follows:

1. We elaborately designed a multiview feature selection model that integrates an autoencoder, multilevel projection learning, consensus dynamic graph learning, and sparse regularization relaxation into a unified framework to select an informative radiomics feature subset from CT, MRI, and US imaging modalities for the robust prediction of outcome of RT treatment for patients with H&N cancers.
2. We explored the inherent associations among CT, QUS, and MRI radiomics features by adaptively learning a consensus Laplacian graph, revealing the shared multilevel projections across different imaging modalities in outcome prediction. The Laplacian graph construction is integrated into an autoencoder to dynamically learn a discriminative and accurate graph structure to obtain the most informative radiomics features. This approach leverages both view‐specific and consensus projections to simultaneously explore global and local structures from heterogeneous features.
3. We introduced a learnable parameter in our model to determine the contribution of each imaging modality in the prediction of treatment outcome.

**FIGURE 1:**
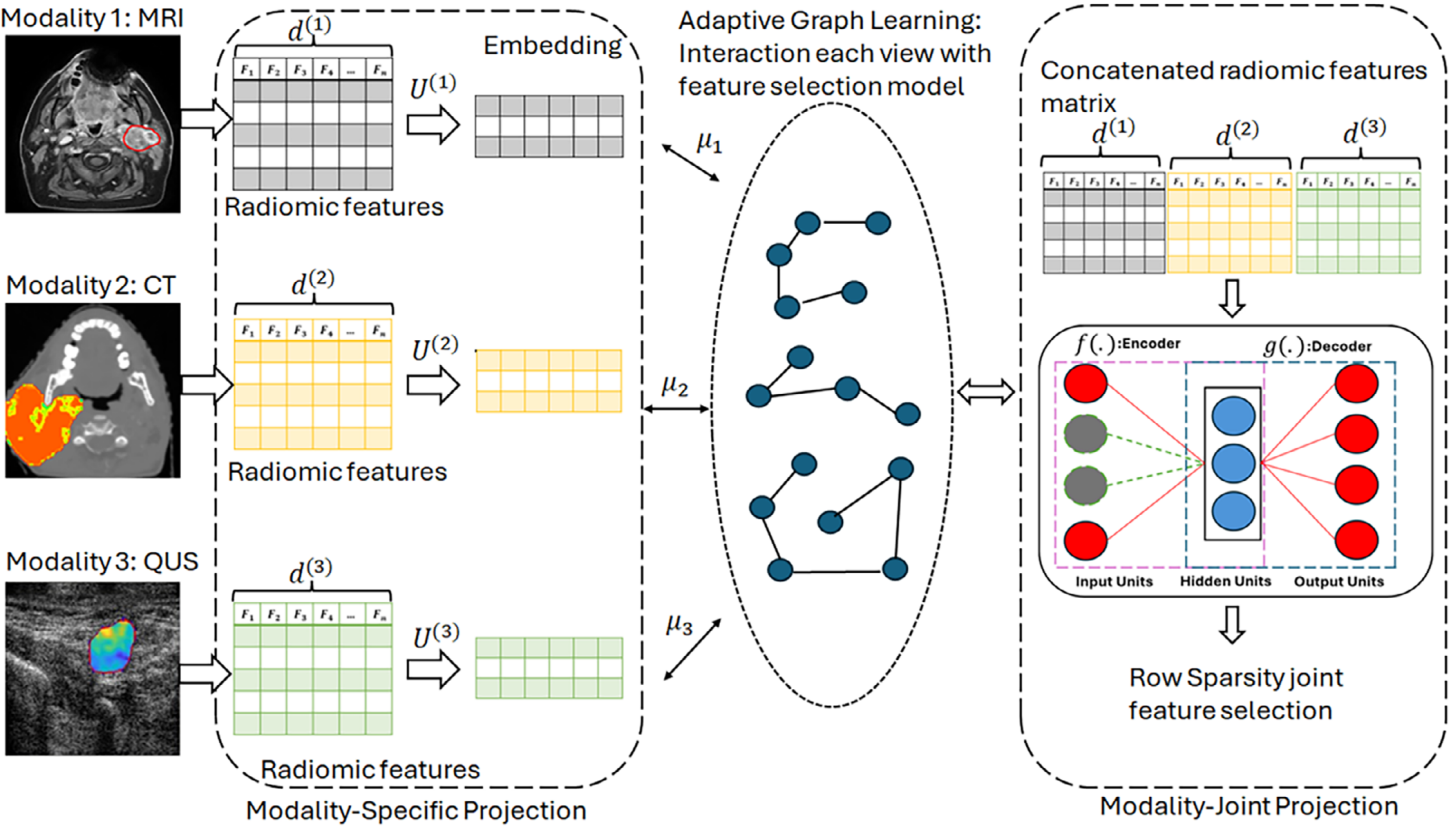
The schematic of the proposed multi‐modal unsupervised feature selection using adaptive graph learning and autoencoder. This technique has two parts: specific projection and joint projection. In specific projection, a projection matrix is learned for each imaging modality and an adaptive neighbor graph is constructed based on these projection matrices. The constructed graph is collaborated with joint projection in order to preserve the geometrical information of data in encoding phase. Where $U^{\left(v\right)}$, $\mu^{\left(v\right)}$ and $d^{\left(v\right)}$ are view specific projection matrix, factor to distinguish different view and extracted radiomic features from view v, respectively.

The steps of proposed MVFS technique are summarized in Figure 1.

## 2 MATERIALS AND METHODS

### 2.1 Study protocol and data acquisition

We obtained approval from the Institutional Research Ethics Board (REB 3047 – Sunnybrook Research Institute) for this study. Patients were informed that they would be considered for research and that there would be no direct benefit or effect on their treatment outcomes. Sixty‐three patients were enrolled and scheduled to receive radiotherapy (XRT). H&N cancers with bulky lymph nodes (LNs) were confirmed by biopsy. US scans were acquired at multiple time points throughout the XRT treatment course. Simulation CT is a crucial preliminary step in nearly all forms of radiotherapy and is used for treatment planning and dosimetric calculations. Maximum and minimum nodal sizes were reported using in‐plane measurements, and index LNs were classified as pathological, and thus potential targets for radiotherapy based on these measurements. According to standard institutional treatment protocols, radical intent radiotherapy, with or without concurrent chemotherapy, was employed to treat patients with H&N cancers. Three different dose levels for RT treatment over 33 fractions were used including 70 Gy as high‐dose, 63 Gy as intermediate‐dose, and 56 Gy prescribed to low‐dose targets. Intensity modulated radiation therapy and volumetric modulation arc therapy as two advanced and common treatment techniques were employed for H&N cancers RT. Additionally, cisplatin, carboplatin, or a combination of both was considered as systemic chemotherapeutics in conjunction with RT.

### 2.2 Data acquisition

#### 2.2.1 Ultrasound

US scans were acquired one week before treatment, as well as 1 day, 1 week, and 4 weeks after treatment was initiated. Each scan lasted between 20 and 45 min. The largest LN was scanned based on radiologist‐dictated patient notes by trained technicians to obtain the whole LN volume along 256 lateral scan lines (3.8 cm lateral field of view and 5 cm maximum axial depth). The depth of each individual's LN was used to set the acoustic focus (average depth of 1.75 cm). A sample rate of 40 MHz was set to collect B‐mode and associated digitized RF signals. The device used was the Ultrasonix Med. Corp. (BC, Canada) with a linear transducer (L14‐5/38 Linear 4D, Ultrasonix) with a central frequency of approximately 7.5 MHz. LN from six equally spaced B‐mode images with associated RF‐data were segmented using MATLAB software. To compute QUS spectra, a Hamming window was applied to individual RF lines before performing a fast Fourier transform (FFT) to determine the frequency components of the RF signal.^14^ In the next step, the power spectrum was divided by the power spectrum of a tissue‐mimicking phantom with known acoustic properties to compute the mean of the squared spectral magnitudes. This was then calibrated to not only remove various frequency‐dependent transfer functions but also eliminate beam‐forming effects associated with the transducer. A line was fitted to normalised power spectrum using linear regression to obtain a fit line within a −5 dB window (bandwidth of 3–8 MHz) centred at the transducer frequency. The parameters mid‐band fit (MBF), spectral slope (SS), spectral intercept (SI) were calculated from the fitted line, and average scatter diameter (ASD), and average acoustic concentration (AAC) parameters from both a Gaussian model and the fluid‐filled model.^14^ Therefore, a sliding window technique with a window block of 2 × 2 mm and a 94.1% overlap between adjacent windows in both axial and lateral directions was applied to seven QUS spectral parameters to generate QUS Parametric maps.

#### 2.2.2 CT

Because CT scanning is essential for planning treatment, participants faced no additional requirements for this part of the research. Data, including treatment planning CT scans, segmentations, and transformation matrices, were obtained from the institutional database in DICOM format. These files were then accessed using the open‐source software 3D Slicer, ensuring precise registration of treatment planning segmentations with the CT image anatomy via the transformation matrix. Target LN regions of interest (ROIs) were identified and saved as .nrrd files, while the CT scans themselves were saved as .nii files. Details regarding the CT imaging and treatment procedures can be found in.^17^ The CT simulator machine was 32‐slice Philips wide‐bore scanner (Philips Brilliance CT Big Bore scanner).CT Bore size was 85 cm and two collimations 16×1.5 and 16×0.75, true field of view was 60 cm with 120 s maximum scan time, 120–140 kV voltage tube and with 80 mili‐liter dose of OMNI350 with injection rate 2cc/s contrast agent. Patients were imaged 45 seconds after injection.

#### 2.2.3 MRI

The main and LN gross tumor volumes were delineated on the planning CT scan, aligned with diagnostic or simulation MRI.^29^ A radiation oncologist with 5 years of experience used pretreatment T1‐weighted post‐contrast MRI to manually segment the region of interest (ROI) using ITK‐SNAP software (Version 3.6.0). The ROI encompassed the largest affected regional lymph node, and if multiple lymph nodes were closely adjacent or formed a conglomerate, all were included within the ROI.

#### 2.2.4 Tumor response definition

Tumor response was evaluated for the primary tumor and regional lymph nodes using contrast‐enhanced MRI at the 3‐month follow‐up after completing radiotherapy. Assessment was based on the response evaluation in solid tumors criteria.^30^ In brief, complete response (CR) was characterized by the disappearance of the primary tumor and a reduction of the involved lymph nodes' short axis to less than 10 mm. Partial response (PR) was defined as at least a 30% decrease in the sum of tumor diameters compared to the baseline MRI. The protocol also outlined the evaluation of patients with stable and progressive disease, but as no instances of these were present in the cohort, they are not detailed further in this methodology.

#### 2.2.5 Radiomic features

LN segmentation ROIs were utilized as masks with corresponding QUS, CT, and MRI scans. To address variations in pixel intensities across scans, images underwent normalization by centering them at the mean and standard deviation of pixel values in the entire image.^31^ Twenty‐four Gray Level Co‐occurrence Matrix (GLCM), 16 Gray Level Run Length Matrix (GLRLM), 16 Gray Level Dependence Matrix (GLDM), and 14 Gray Level Size Zone Matrix (GLSZM) 2D texture features were extracted from CT and MRI for each modality. For QUS, these 70 radiomics features were determined for each of the QUS parameters, including MBF, SS, SI, ASD, and AAC. Therefore, QUS had 350 radiomic features, CT has 70 radiomic features, and MRI has 70 radiomic features. Radiomic features were determined using a Pyradiomics Python package.^31^

### 2.3 Multiview feature selection

#### 2.3.1 Notation

In this study, matrix and vector were shown by capital letter (e.g., X) and small letter (e.g., x), respectively. $X^{T}$ shows the transpose and Tr(X) is the trace of matrix X (sum of diagonal elements). 1 and I represent a vector whose elements are all ones, and the identity matrix, respectively. ${∥X∥}_{F}^{2}=\sum_{ij}x_{ij}^{2}$ and $∥X{∥}_{p,q}={\left(\sum_{i}{∥x_{i}∥}_{p}^{q/p}\right)}^{1/q}$ are squared Frobenius norm and $l_{p,q}$‐norm, respectively. $∥x{∥}_{0}$ is the number of nonzero elements. In our study, we had three imaging modalities and thus V=3. Summary of the key notations used in the paper is listed in Table 1.

**TABLE 1:** Summary of the key notations used in the paper.

Notation	Explanations
$X^{\left(v\right)}\in R^{n\times d_{v}}$	Data in view v, with n samples and $d_{v}$ features
$\bar{X}\in R^{n\times M}$	The concatenation of all views. M is number of features for all views.
$W^{\left(1\right)}\in R^{M\times k_{w}}$	The encoder weight matrix, $k_{w}$ is dimension of hidden layer.
$W^{\left(2\right)}\in R^{k_{w}\times M}$	The decoder weight matrix
$U^{\left(v\right)}\in R^{d_{v}\times k_{v}}$	The projection matrix for each view (imaging modalities).
$S\in R^{n\times n}$	Affinity matrix
V= 3	Total number of views (imaging modalities).
k	The number of nearest neighbors to a sample.
$s_{ij}$	Element ij in similarity matrix
D	Diagonal degree matrix
L=D−S	Laplacian matrix
$||w_{i}^{\left(1\right)}|{|}_{2}^{2}$	Feature score
μ	Weight factor to distinguish different views
β	Regularization coefficient of group LASSO regularization
α	Weight decay coefficient
η	Regularization coefficient to avoid trivial solution
θ	Trade‐off regularization parameter between structure learning Regularization and error reconstruction loss,
γ	Parameter to avoid trivial case

## 3 BACKGROUND

Dimensionality reduction is a crucial step in data preprocessing aimed at addressing the curse of dimensionality and overfitting. Feature extraction and feature selection are two primary approaches used for reducing dimensionality. Feature extraction techniques such as principal component analysis (PCA),^32^ linear discriminant analysis (LDA),^33^ independent component analysis,^34^ canonical correlation analysis,^35^ and autoencoder^36^ are applied to reduce dimensionality but typically lack interpretability. In contrast, feature selection methods possess the ability to identify the most informative and discriminative features.

Feature selection techniques are categorized based on label availability and selection strategy.^37^ Supervised feature selection uses label information to rank features, examples include mRMR.^38^ Unsupervised feature selection ranks features based on information and dependencies among features; nonnegative matrix factorization (NMF) is a well‐known technique in this category.^39^ Semi‐supervised feature selection addresses scenarios where only part of the data is labeled, commonly employing methods such as Hessian‐based and Laplacian‐based approaches.^40^

In terms of strategy, feature selection techniques are classified into filter, wrapper, embedded and hybrid strategies.^41^ In the filter strategy, features are selected independently of any classifier. The wrapper strategy selects features based on the performance of a classifier, while embedded strategies incorporate feature selection as part of the training process, such as in least absolute shrinkage and selection operator (LASSO) regression and decision trees. Hybrid feature selection methods combine multiple strategies, such as filter and wrapper approaches.^37^

However, implementing feature selection becomes challenging when data is sourced from diverse origins or described in different formats. For example, in,^42^ features were extracted from inspiratory lung CT, expiratory lung CT, and the registration of expiratory and inspiratory lung CT. These features were concatenated and subjected to NMF‐based feature selection, despite originating from three distinct types. Feature selection for features with different sources follows three types of pipeline. The first pipeline, which is very common, is to concatenate all features and consider the problem as a single‐source feature selection.^43^ The first pipeline, concatenation of all features from different modalities (views), neglects the differences among sources (modalities). The second pipeline considers each feature source separately and applies optimization independently to each source (view).^44^ Although the second pipeline does not discard the differences among feature sources, it ignores the associations between different sources.

The final pipeline is MVFS, which successfully performs joint selection of heterogeneous features, a capability that neither the first nor the second pipeline possesses. MVFS represents a branch of machine learning dedicated to managing the heterogeneous nature of multiview or multi‐modal imaging features.^45^, ^46^ Traditional techniques for multi‐modal imaging typically employ single‐view feature selection by aggregating all features, which is simplistic and fails to capture inter‐view correlations. However, preserving such correlations is crucial for effective feature selection.^37^ MVFS integrates inter‐view correlations into the feature selection algorithm to extract a consensus data embedding based on collaborative views.^47^, ^48^

Unsupervised feature selection utilizes inherent data embeddings to guide initial feature evaluations. Many methods leverage spectral analysis on graph structures for graph embedding, benefiting from the robust representational power of graphs. For instance, Tang et al.^49^ introduced an unsupervised MVFS technique based on graph representation learning. This approach constructs a Laplacian graph matrix for each view independently and defines a consensus embedding space to project each view. The objective function of this technique is formulated as follows:

(1) $$\begin{array}{cc} & \min_{Z,W^{v}}\sum_{v=1}^{V}\lambda_{v}Tr\left(Z^{T}L_{v}Z\right)+\alpha ∥X^{T^{\left(v\right)}}W^{v}\\ & {\;-Z∥}_{F}^{2}+\beta ∥W{∥}_{2,1}\;s.t\;Z^{T}Z=I,\;Z≽0\; \end{array}$$

where $X^{v}$, Z, L, and W represent data in view v, consensus embedding, Laplacian graph matrix and projection matrix, respectively. However, there are two primary concerns regarding this MVFS technique:

- The correlation among view‐wise projections is ignored, thereby failing to preserve inter‐view feature correlations.
- A fixed similarity matrix is constructed via an optimization problem, potentially reducing the effectiveness of feature selection due to noise and outliers.

In the MVFS strategy, collaboration among views (imaging modalities) is considered not only to extract the consensus embedding of the data but also to learn view‐specific projections guided by this consensus embedding.

### 3.1 Proposed MVFS

Spectral analysis‐based unsupervised feature selection methods have received significant attention for their ability to preserve the manifold structure of data in a low‐dimensional subspace.^50^ The construction of an affinity graph plays a significant role in spectral analysis‐based techniques, and incorporating adaptive graph learning enhances robustness against outliers and noisy data. Additionally, autoencoder has recently indicated considerable strength in features selection due to their ability to extract nonlinear patterns.^51^ Therefore, we applied adaptive graph learning in combination with an autoencoder in our proposed unsupervised feature selection method.

We proposed a MVFS technique named Adaptive Graph Autoencoder Multi‐View Feature Selection (AGAMVFS), based on dynamic graph learning and autoencoder. In AGAMVFS, adaptive and collaborative graphs are learned at multiple levels to discriminate among view‐specific features. An autoencoder is then applied to concatenated features to select the most discriminative ones. This approach fosters collaboration across different views and achieves a consensus projection for feature selection.

#### 3.1.1 AGAMVFS formulation

AGAMVFS contains two parts including all‐views and each‐view specific. An autoencoder was used for all‐views part to apply on all concatenated features and a projection matrix was considered for each‐view specific.

**Part 1: All views**

For feature selection, a robust autoencoder feature selection technique was utilized. An autoencoder is categorized as a neural network or deep learning model, which can be trained to learn patterns in data with the aim of reconstructing its input using a series of nonlinear transformations. In an autoencoder, compressed latent representations of the data can be learned by enforcing the reconstructed data to be close to its input. Autoencoder can capture the essential structure of the data necessary for reconstruction.^52^ In the context of feature selection, Ling et al.^51^ experimentally showed that selected features by the autoencoder efficiently preserve substantial information for the subsequent clustering goal. Generally, autoencoder has two parts including the encoding and the decoding. $W^{\left(1\right)}$ and $W^{\left(2\right)}$ are the synaptic weights of encoding phase and decoding phase, respectively. Input data X is projected into latent embedding space by $W^{\left(1\right)}$ ($XW^{\left(1\right)}$) and it is reconstructed by $W^{\left(2\right)}$. Autoencoder network is trained using the following mean squared error function between the input data and the reconstructed data:

(2) $$\min_{W^{\left(1\right)},W^{\left(2\right)}}\frac{1}{2n}{∥X-g\left(f\left(X\right)\right)∥}_{F}^{2}$$

where $f\;\left(X\right)=\sigma \left(XW^{\left(1\right)}\right)$, $g\;\left(f(X)\right)=\sigma \left(f\left(X\right)\;W^{\left(2\right)}\right)$, and σ is sigmoid activation function. $W^{\left(1\right)}$ maps the input data to a compressed latent space, allowing the measurement of feature importance within $W^{\left(1\right)}$. $W^{\left(1\right)}={\left[w_{1},w_{2},\text{…},w_{d}\right]}^{T}$, where the i‐th row of $W^{\left(1\right)}$ is $w_{i}$ and shows the importance of i‐th feature in the input data X. The contribution of each feature is measured by $∥w_{i}{∥}_{2}$, and $∥w_{i}{∥}_{2}\approx 0$ indicates that the i‐th feature in the input data X is redundant and makes a negligible contribution to the representation of other features.

On the other hand, if $∥w_{i}{∥}_{2}$ is significant, the i ‐th feature is important and plays a significant role in the representation of other features. Therefore, we applied the mixed‐norm $∥W{∥}_{2,1}$ as a row‐sparse regularization function to preserve the global information of the data. Additionally, ridge regularization (weight decay) $∥W{∥}_{F}$ was considered to avoid overfitting and promote convergence. In addition to preserving global information, local structural learning must be applied to preserve the geometry of data. To better understand local structure learning; if two samples $x_{1}$ and $x_{2}$ in the input data X are close, then the corresponding projected samples $W^{{\left(1\right)}^{T}}x_{1}$ and $W^{{\left(1\right)}^{T}}x_{2}$ must also be close.^37^ Therefore, the Laplacian graph matrix was applied to preserve the geometrical information of data. To this end, ${∥X∥}_{M}^{2}=\int ∥\nabla_{M}F{∥}^{2}$ is defined to measure the smoothness of F along the geodesic on an unknown submanifold $M\subset R^{m}$ ($\nabla_{M}$ F is the gradient of input data X with submanifold M).^37^ Since M is unknown the continuous ${∥F∥}_{M}^{2}$ cannot be computed and it can be discretely approximated as follows:

(3) $$\begin{array}{cc} \; & \min_{W}\sum_{i,j}{∥W^{T}x_{i}-W^{T}x_{j}∥}_{2}^{2}s_{ij}\\ & \;=\sum_{i=1}^{n}{\left(W^{T}x_{i}\right)}^{T}W^{T}x_{i}D_{ii}-\sum_{i,j=1}^{n}{\left(W^{T}x_{i}\right)}^{T}W^{T}x_{j}s_{ij}\\ & \;=Tr\left(X^{T}W^{T}DXW\right)-Tr\left(X^{T}W^{T}SXW\right)\\ & \;=Tr\left(W^{T}X^{T}LXW\right)\; \end{array}$$

where $D_{ii}=\sum_{j=1}^{n}s_{ij}$ and L=D−S are diagonal matrix, whose diagonal elements are sum of S‐row, and Laplacian matrix, respectively. D is degree matrix and S is affinity matrix. In AGAMVFS, S is updated in each iteration and adapts to learn from data representations, where a large similarity indicates similar representations of samples.

**FIGURE 2:**
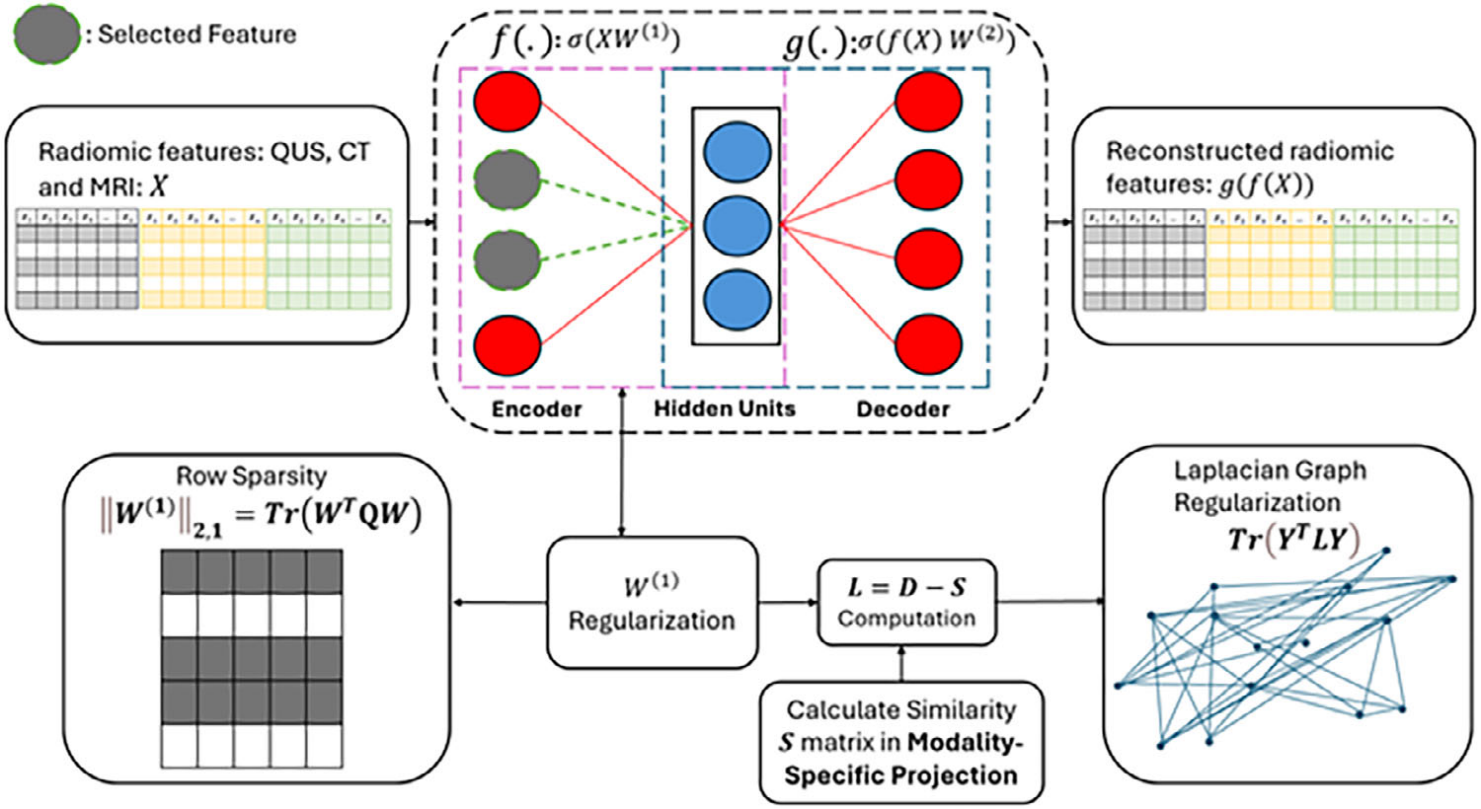
The schematic of feature selection using autoencoder in the Modality‐Joint phase (all‐views) is as follows: Features from all modalities are concatenated and then fed into the autoencoder for the purpose of feature selection.. In each iteration, the Laplacian graph is updated using the similarity matrix in the modality‐specific projection phase. Where $Q=diag(\frac{1}{2∥w_{i}^{\left(1\right)}{∥}_{2}+\varepsilon })$ and ε is small value to avoid instability.

Finally, we have objective function with all regularization functions as follows:

(4) $$\begin{array}{cc} & \min_{W^{\left(1\right)},W^{\left(2\right)}}\frac{1}{2n}{∥X-g\left(f\left(X\right)\right)∥}_{F}^{2}+\frac{\theta }{2}Tr\left(Y^{T}LY\right)\\ & \;+\frac{\beta }{2}∥W^{1}{∥}_{2,1}+\frac{\alpha }{2}\sum_{i=1}^{2}{∥W^{\left(i\right)}∥}_{F}^{2} \end{array}$$

where $Y=\sigma (XW^{1})$, and θ, β and α are regularization coefficients. Figure 2 shows the feature selection using autoencoder.

**Part 2: Each View Specific**

The original feature space contains redundant and irrelevant features that degrade the performance of feature selection. Adaptive affinity matrices improve learning and enhance the performance of feature selection. Nei et al.^53^ proposed Structured Graph Optimization (SGO), which is an adaptive graph learning technique for single‐view data feature selection. In AGAMVFS, we employed the SGO strategy for multiview data, considering that information from multiple views is complementary yet exhibits a coherent underlying cluster structure. Therefore, multilevel projections with dynamic collaborative neighbor graph learning for different views are expressed as follows:

(5) $$\begin{array}{c} \begin{array}{c} \min_{\mu ,U,S}\left(\sum_{v=1}^{3}\mu_{v}^{\gamma }\sum_{i,j}\left(∥U^{{\left(v\right)}^{T}}x_{i}^{\left(v\right)}-U^{{\left(v\right)}^{T}}x_{j}^{\left(v\right)}{∥}_{2}^{2}s_{i,j}\right)\right)+\eta \sum_{i,j}s_{ij}^{2}\\ s.t.\;U^{{\left(v\right)}^{T}}\;U^{\left(v\right)}=I,\;\mu^{T}1=1,\mu \geq 0,\;s_{i,:}^{T}1=1,\;s_{i,j}\geq 0,\;\;∥s_{i,:}{∥}_{0}=k \end{array} \end{array}$$

where $S\in R^{n\times n}$, $U^{\left(v\right)}\in R^{d_{v}\times k_{v}}$, $X^{\left(v\right)}\in R^{n\times d_{v}}$ and $\mu \in R^{v}$ are affinity matrix, view specific projection matrix, view specific input data and weight factor to distinguish different views, respectively. n is the number of samples and $d_{v}$ is the number of features in view v. S is optimized cross all views to update Laplacian graph matrix. Concretely, $x_{j}$ is the neighbor of $x_{i}$ with probability $\;s_{i,j}$ and the smaller value of distance $∥U^{{\left(v\right)}^{T}}x_{i}^{\left(v\right)}-U^{{\left(v\right)}^{T}}x_{j}^{\left(v\right)}{∥}_{2}^{2}$, the larger the probability corresponds. Additionally, $\sum_{i,j}s_{ij}^{2}$ term controls the solution to avoid a trivial solution. $s_{i,:}^{T}1=1$ constraint guarantees that $0\leq s_{i,j}\leq 1$ (1 is n ‐dimensional column Vector), since $s_{i,j}$ is the probability of connection between $x_{j}$ and $x_{i}$. $U^{\left(v\right)}$ is an orthonormal matrix to project $X^{\left(v\right)}$ into latent space to extract discriminative embedding from view‐specific features and $U^{{\left(v\right)}^{T}}\;U^{\left(v\right)}=I$ is the orthogonality constraint.

γ>1 is a parameter to avoid trivial case (a case where the best view is only determined with nonzero weight) and η is regularization coefficient to avoid trivial solution, respectively. Learning similarity relation from the k‐nearest projected neighbors is more accurate than learning from the original representation.^54^ We considered a weight (μ) for each view in order to preserve the correlation among views and distinguish heterogeneous features. μ is an adaptive parameter that is updated and learned in each iteration. The subtle point should be mentioned is that the weight of each view is obtained based on cut value (e.g., small cut value is allocated with a large weight). The similarity matrix, view‐weight, and view specific projection are jointly optimized to achieve maximum differences of samples between clusters and minimum differences of samples within cluster. In each iteration, the similarity matrix S is constructed and subsequently Laplacian graph matrix is updated. We integrated model (5) into model (4) to build AGAMVFS. In AGAMVFS, we have contribution of both view specific representations ($X^{\left(v\right)}\in R^{n\times d_{v}})$ and the concatenated representations ($\bar{X}$) to construct and update the matrix S, thereby capturing both within‐view and cross‐view information.

Therefore, the final objective function AGAMVFS, which encompasses view‐specific data and concatenated data, is formulated as follows:

(6) $$\begin{array}{cc} & \min_{W^{\left(1\right)},W^{\left(2\right)},\mu ,U,S}\;\omega \left(\frac{1}{2n}{∥X-g\left(f\left(X\right)\right)∥}_{F}^{2}\right.\\ & \;\left.+\frac{\theta }{2}Tr\left(Y^{T}LY\right)+\frac{\beta }{2}∥W^{1}{∥}_{2,1}+\frac{\alpha }{2}\sum_{i=1}^{2}{∥W^{\left(i\right)}∥}_{F}^{2}\right)\\ & \;+\sum_{v=1}^{3}\mu_{v}^{\gamma }\sum_{i,j}(∥U^{{\left(v\right)}^{T}}x_{i}^{\left(v\right)}-U^{{\left(v\right)}^{T}}x_{j}^{\left(v\right)}{∥}_{2}^{2}s_{i,j})+\eta \sum_{i,j}s_{ij}^{2}\\ & s.t.\;U^{{\left(v\right)}^{T}}\;U^{\left(v\right)}=I,\;\mu^{T}1=1,\mu \geq 0,\;s_{i,:}^{T}1=1,\\ & \;s_{i,j}\geq 0,\;\;∥s_{i,:}{∥}_{0}=k \end{array}$$

where ω is a coefficient to trade‐off between view‐within information and cross‐view information. While the diverse characteristics demonstrate the consistent clustering pattern within the data, it is important to acknowledge that features across various views may provide complementary knowledge. AGAMVFS aims to integrate discriminative view‐specific projections with indiscriminate joint projections, enhancing the efficacy of crucial views while mitigating the loss of information from less informative views. Additionally, employing multilevel similarity learning diminishes the influence of single‐view projections on similarity determination and reinforces the stability of graph learning.

#### 3.1.2 Optimization of AGAMVFS

We employed block coordinate descent method^55^ to update each variable when other variables fixed.

**Update** S **with the fixed** U, μ, $W^{\left(1\right)}$ **and** $W^{\left(2\right)}$

The problem (6) is reformulated to (7) when U, μ, $W^{\left(1\right)}$ and $W^{\left(2\right)}$ are fixed.

(7) $$\begin{array}{c} \begin{array}{c} \min_{S}\sum_{v=1}^{3}\mu_{v}^{\gamma }\sum_{i,j}(∥U^{{\left(v\right)}^{T}}x_{i}^{\left(v\right)}-U^{{\left(v\right)}^{T}}x_{j}^{\left(v\right)}{∥}_{2}^{2}s_{i,j})+\eta \sum_{i,j}s_{ij}^{2}\\ \;+\frac{\omega \theta }{2}Tr\left(Y^{T}LY\right)\\ s.t.\;s_{i,:}^{T}1=1,\;s_{i,j}\geq 0,\;\;∥s_{i,:}{∥}_{0}=k \end{array} \end{array}$$

From spectral analysis (3) we know that

(8) $$Tr\;\left(Y^{T}LY\right)=\frac{1}{2}\;\sum_{i,j}{∥y_{i}-y_{j}∥}_{2}^{2}s_{ij}$$

Let we define $z_{ij}=(\sum_{v}∥U^{{\left(v\right)}^{T}}x_{i}^{\left(v\right)}-U^{{\left(v\right)}^{T}}x_{j}^{\left(v\right)}{∥}_{2}^{2})+\frac{\omega \theta }{4}\sum_{i,j}{∥y_{i}-y_{j}∥}_{2}^{2}$, and we reformulate (7) as follows:

(9) $$\min_{S}\sum_{i,j}\left(z_{ij}s_{ij}+\eta s_{ij}^{2}\right)\;s.t.\;s_{i,:}^{T}1=1,\;s_{i,j}\geq 0,\;\;∥s_{i,:}{∥}_{0}=k$$

We know that ${\left(z_{ij}+\eta s_{ij}\right)}^{2}=z_{ij}^{2}+2\eta s_{ij}z_{ij}+\eta^{2}s_{ij}^{2}$, and rows of matrix S ($s_{i,:}$) are independent of each other; therefore we can reformulate optimization problem for each row as follows:

(10) $$\min_{s^{i}}∥s_{i,:}+\frac{z_{i,:}}{2\eta }{∥}_{2}^{2}s.t.\;s_{i,:}^{T}1=1,\;s_{i,j}\geq 0,\;\;∥s_{i,:}{∥}_{0}=k$$

$\;∥s_{i,:}{∥}_{0}=k$ represents that each row of S ($s_{i,:})$ has exactly k nonzero value. Based on (10), the minimization problem (10) would be more decreased by the smaller value of $z_{i,:}$ when $s_{ij}=0$. Therefore, $z_{i,:}$ is sorted in ascending form $z_{i,1}\leq z_{i,2}\leq \text{…}\leq z_{i,n}$. To apply $∥s_{i,:}{∥}_{0}=k$ constraint, we consider n−k elements of vector $s_{i,:}$ zero and update only k elements of vector $\;s_{i,:}$ in each iteration which leads to a reduction in computational complexity. Therefore, each element of similarity matrix S is update as follows:

(11) $$\;s_{ij}=T\left(\frac{z_{i,k+1}-z_{ij}}{kz_{i,k+1}-\sum_{j=1}^{k}z_{ij}}\right)$$

where function T is defined as follows:

(12) $$T\;\left(x\right)=\left\{\begin{array}{c} x,\;if\;x\geq 0\\ 0,\;if\;x<0 \end{array}\right.\;$$

The details of mathematical derivation for $s_{ij}$ are presented in Appendix A.

**Update** μ **with the fixed** U, S, $W^{\left(1\right)}$ **and** $W^{\left(2\right)}$

The optimization problem for variable μ is reformulated as follows:

(13) $$\min_{\mu }\sum_{v=1}^{3}\mu_{v}^{\gamma }E^{v}\;s.t.\;\mu^{T}1=1,\mu_{v}\geq 0$$

where $E^{\left(v\right)}=\sum_{i=1}^{n}\sum_{j=1}^{n}{∥U^{{\left(v\right)}^{T}}x_{i}^{\left(v\right)}-U^{{\left(v\right)}^{T}}x_{j}^{\left(v\right)}∥}_{2}^{2}s_{i,j}$. Parameter $E^{\left(v\right)}$ obtains the differences withing v‐th view. The variable μ is updated in each iteration as follows:

(14) $$\;\mu_{v}=\frac{{\left(E^{\left(v\right)}\right)}^{\frac{1}{1-\gamma }}}{\sum_{v=1}^{3}{\left(E^{\left(v\right)}\right)}^{\frac{1}{1-\gamma }}}\;$$

The details of mathematical derivation for μ are presented in Appendix B.

**Update** U **with the fixed** μ, S, $W^{\left(1\right)}$ **and** $W^{\left(2\right)}$

The objective function to update U is formulated as follows:

(15) $$\min_{U}\sum_{v=1}^{3}\mu_{v}^{\gamma }\sum_{i,j}(∥U^{{\left(v\right)}^{T}}x_{i}^{\left(v\right)}-U^{{\left(v\right)}^{T}}x_{j}^{\left(v\right)}{∥}_{2}^{2}s_{i,j}\;s.t.\;U^{{\left(v\right)}^{T}}\;U^{\left(v\right)}=I\;$$

From spectral analysis (3) we have

(16) $$\min_{U}\sum_{v=1}^{3}Tr(U^{{\left(v\right)}^{T}}\left(\mu_{v}^{\gamma }X^{\left(v\right)}L_{S}X^{{\left(v\right)}^{T}}\right)U^{\left(v\right)}\;s.t.\;U^{{\left(v\right)}^{T}}\;U^{\left(v\right)}=I$$

The projection matrix $U^{\left(v\right)}$ is independent of each other, then we can solve (16) for each view separately. (16) is an eigen problem and the solution of $U^{\left(v\right)}\in R^{d_{v}\times k_{v}}$ is the $k_{v}$ eigenvectors of $\mu_{v}^{\gamma }X^{\left(v\right)}LX^{{\left(v\right)}^{T}}$ corresponding to its $k_{v}$ smallest eigenvalues.

**Update** $W^{\left(1\right)}$ **and** $W^{\left(2\right)}$ **with the fixed** μ, S, U

To obtain the solutions of $W^{\left(1\right)}$ and $W^{\left(2\right)}$, we have following objective function:

(17) $$\begin{array}{cc} F\;\left(W\right) & =\min_{W^{\left(1\right)},W^{\left(2\right)}}\;\frac{1}{2n}{∥\bar{X}-g\left(f\left(X\right)\right)∥}_{F}^{2}+\frac{\theta }{2}Tr\left(Y^{T}LY\right)\\ & \;+\frac{\beta }{2}∥W^{\left(1\right)}{∥}_{2,1}+\frac{\alpha }{2}\sum_{i=1}^{2}{∥W^{\left(i\right)}∥}_{F}^{2} \end{array}$$

where $\bar{X}$ is concatenation of all features. To solve (17) and update $W^{\left(1\right)}$ and $W^{\left(2\right)}$, we employed back‐propagation strategy, limited‐memory BFGS (L‐BFGS)^56^ and minFunc^57^ toolbox. Let show reconstructed data by $X^{\prime }=g\left(f(X)\right)$, then the error terms of output layer and hidden layer are obtained as follows:

(18) $$\delta^{\left(o\right)}=-\left(X-X^{\prime }\right)⊙\sigma^{\prime }\left(f\left(X\right)\right)\;\;\;\;\;\;\text{Output}\;\;\;\;\;\;\text{layer}$$

(19) $$\delta^{\left(h\right)}=\left(W^{{\left(2\right)}^{T}}\delta^{\left(o\right)}\right)⊙\sigma^{\prime }\left(X\right)\;\;\;\;\;\;\text{Hidden}\;\;\;\;\;\;\text{layer}$$

where ⊙ shows the Hadamard product (element‐wise product). Let we define $∥W^{\left(1\right)}{∥}_{2,1}=Tr\left(W^{{\left(1\right)}^{T}}QW^{\left(1\right)}\right)$, where $Q=diag(\frac{1}{2∥w_{i}^{\left(1\right)}{∥}_{2}+\varepsilon })$ and ε is small value to avoid instability. Therefore, we showed the partial derivative of (25) with respect to $W^{\left(1\right)}$ and $W^{\left(2\right)}$ in (20) and (21), respectively.

(20) $$\;\frac{\partial F\left(W\right)}{\partial W^{\left(1\right)}}=X{\left(\delta^{\left(h\right)}\right)}^{T}+\beta QW^{\left(1\right)}+\alpha W^{\left(1\right)}+\theta LY$$

(21) $$\;\frac{\partial F\left(W\right)}{\partial W^{\left(2\right)}}=f\left(x\right){\left(\delta^{\left(o\right)}\right)}^{T}+\alpha W^{\left(2\right)}$$

Then update rule of $W^{\left(1\right)}$ and $W^{\left(2\right)}$ are as follows:

(22) $$W^{\left(1\right)}\leftarrow W^{\left(1\right)}-\xi_{1}\frac{\partial F\left(W\right)}{\partial W^{\left(1\right)}}$$

(23) $$W^{\left(2\right)}\leftarrow W^{\left(2\right)}-\xi_{2}\frac{\partial F\left(W\right)}{\partial W^{\left(2\right)}}$$

where $\xi_{1}$ and $\xi_{2}$ are learning rates (step‐size).

The AGAMVFS technique is summarized in Algorithm 1.

ALGORITHM 1 **Algorithm to solve AGAMVFS technique.**

**Table:** 

**Input**: Different modalities data $X={\left\{X^{\left(v\right)}\right\}}_{v=1}^{3}$, where $X^{\left(v\right)}\in R^{n\times d_{v}}$, n is the number of sample (patient) and $d_{v}$ is the number of features for each view. Parameters ω, θ, β, α, and γ. $M=d_{1}+d_{2}+d_{3}$ is the number of features for all views (We have three imaging modalities including QUS, CT, and MRI).	
**Initialize**: The random orthogonal projections ${\left\{U^{\left(v\right)}\right\}}_{v=1}^{3}\in R^{d_{v}\times k_{v}}$ which satisfying $U^{{\left(v\right)}^{T}}\;U^{\left(v\right)}=I$ and $k_{v}$ is the reduced dimension for each modality; randomly initialize $W^{\left(1\right)}\in R^{M\times k_{w}}$ and $W^{\left(2\right)}\in R^{k_{w}\times M}$; $Q=I\in R^{M\times M}$, where I is identity matrix; the similarity matrix $S\in R^{n\times n}$ are zero matrix; the weights for each views as $\mu_{v}=\frac{1}{V}$, where V=3 (the number of modalities).	
**for** iteration =1 to t **do**	
1‐	For each row‐ i, compute $z_{ij}=(\sum_{v}∥U^{{\left(v\right)}^{T}}x_{i}^{\left(v\right)}-U^{{\left(v\right)}^{T}}x_{j}^{\left(v\right)}{∥}_{2}^{2})+\frac{\omega \theta }{4}\sum_{i,j}{∥y_{i}-y_{j}∥}_{2}^{2}$, where j is the k‐nearest neighbor, and therefore update each row‐ i using Equation (11) to obtain similarity matrix S;
2‐	Compute Laplacian matrix L=D−S, where $D=diag(\sum_{j}S^{ij}$) is degree matrix.
3‐	For each modality v, compute $E^{\left(v\right)}=\sum_{i=1}^{n}\sum_{j=1}^{n}{∥U^{{\left(v\right)}^{T}}x_{i}^{\left(v\right)}-U^{{\left(v\right)}^{T}}x_{j}^{\left(v\right)}∥}_{2}^{2}s_{i,j}$ and update $\mu_{v}$ using Equation (14);
4‐	For each modality v, update $U^{\left(v\right)}$ by eigenvectors of $\mu_{v}^{\gamma }X^{\left(v\right)}LX^{{\left(v\right)}^{T}}$ corresponding to its $k_{v}$ smallest eigenvalues;
5‐	Compute $\delta^{\left(o\right)}$ using Equation (18), and $\delta^{\left(h\right)}$ using Equation (19);
6‐	Update $W^{\left(1\right)}$ by Equation (22);
7‐	Update $W^{\left(2\right)}$ by Equation (23);
8‐	Update Q by $Q=diag(\frac{1}{2∥w_{i}^{\left(1\right)}{∥}_{2}+\varepsilon })$;
**end**	
**Output**: Calculate the feature score $||w_{i}^{\left(1\right)}|{|}_{2}^{2}$, ($i=1,2,\text{…},d_{v})$, and sort features based on its score. Select top r‐feature after ranking features.

### 3.2 Computation complexity

In this section, we analyzed computational complexity of each variable of AGAMVFS.

Variable S:

To compute S, we need to compute $z_{ij}$ with computational complexity $O(n^{2}M+n^{2}\log n)$. Due to constraint $∥s_{i,:}{∥}_{0}=k$, only k elements of $s_{i,:}$ are updated in each iteration. k elements of $s_{i,:}$, which show the distance between each sample and its k nearest neighbors, are updated by latest $U^{\left(v\right)}$ and $W^{\left(1\right)}$ which takes the computational complexity of $O(nM^{2}k_{w}t+nkt)$ where t is the number of iterations. The computational complexity of S is $O(nM^{2}k_{w}t+nkt+n^{2}M+n^{2}\log n)$.

Variable $\mu_{v}$:

To compute $\mu_{v}$, we need to compute $E^{\left(v\right)}$ for each view. updating $\mu_{v}$ at each iteration for each view takes the computational complexity of O(nVk).

Variable U:

To compute $\mu_{v}^{\gamma }X^{\left(v\right)}LX^{{\left(v\right)}^{T}}$, the computational complexity is $O(n^{2}d_{v}+nd_{v}^{2})$ and takes $O(d_{v}^{2}k_{v})$ to obtain $k_{v}$ eigenvectors of $\mu_{v}^{\gamma }X^{\left(v\right)}LX^{{\left(v\right)}^{T}}$. Therefore, to update for all views U, the computational complexity is $O(\sum_{v}\left(n^{2}d_{v}+nd_{v}^{2}+d_{v}^{2}k_{v}\right))$.

Variables $W^{\left(1\right)}$ and $W^{\left(2\right)}$:

The computational complexity for $W^{\left(1\right)}$ and $W^{\left(2\right)}$ at each iteration are $O(k_{w}Mn+M^{2}k_{w})$ and $O(k_{w}Mn+Mn^{2}$), respectively.

### 3.3 Classifier training

We employed SVM and KNN classifiers to train a predictive model to discriminate between CR and PR. The AGAMVFS is a filter‐based strategy feature selection, where features are first selected and then used to train the classifier. We applied grid search algorithm to optimize the hyperparameters of classifiers. The parameter “k” is the only hyperparameter of KNN classifiers which assigns the number of neighbors. SVM classifier has hyperparameters “C” and gamma. Parameter “C” controls the trade‐off between the number of nondeferrable samples and complexity of hyperplane, and parameter gamma is used to control the radius of radial basis function kernel. Specificity, sensitivity, accuracy, F1‐score, and balanced‐accuracy (B‐ACC) were used as classification metrics to assess the performance of the proposed method.

### 3.4 Parameter settings

In AGAMVFS, θ, β, α, and η are hyperparameters. θ is a trade‐off regularization parameter between structure learning regularization and error reconstruction loss, and β is regularization coefficient of group LASSO regularization. θ and β are searched in the grid of $\left\{10^{-3},10^{-2},\text{…},10^{2},10^{3}\right\}$. Parameter η is heuristically determined as explained in (A7) Appendix A. Weight decay coefficient α is searched in the grid of $\left\{10^{-4},10^{-3},10^{-2},and\;10^{-1}\right\}$.

### 3.5 Data preprocessing

Leave‐one‐patient‐out methodology was applied to split data into a training set and test‐set. Training data was normalized using the z‐score ($\frac{F_{i}-mean\left\{F_{i}\right\}}{std}$ where $F_{i}$ is i‐th feature and std is standard deviation of $F_{i}$) to have data with zero mean and 1 standard deviation. The synthetic minority oversampling technique was utilized to address the challenge of imbalanced data. SMOTE was applied only on the training set. Additionally, five‐fold cross‐validation was applied on training set to tune hyper parameter of SVM and KNN (Searched over “C” ε{0.1, 1, 10, 100} and “gamma”ε{0.001, 0.01, 0.1, 1}, for KNN, searched over the number of neighbors k∈{3, 5, 7, 9, 11} using Euclidean distance as the metric.).

### 3.6 Statistical test

We performed a two‐sample *t*‐test on the top selected features to assess their statistical significance. p‐value was used to define the significance of the statistical test.

All methods are implemented in the MATLAB 2020b (Version 9.9.0.1592791 (R2020b)) and conducted on the machine with an Intel(R) Core(TM) i7‐ 1065G7 1.50GHZ CPU, and 16GB RAM.

## 4 RESULTS

### 4.1 Patient characteristics

In this study, we had a total of 63 patients with H&N cancers. The average age of patients was 61 years (at the time of diagnosis) and 94% were male. 94% of tumors (*n* = 59) were squamous cell carcinomas and the location of primary tumors were in the oropharynx (68%, n=43), larynx (6%, n=4), hypopharynx (5%, n=3), and nasopharynx (8%, *n* = 5). 13% (*n *= 8) of tumors were unspecified as primary locations. 54 patients received platin‐based concurrent chemotherapy. 23 patients had CR and 40 patients had PR 3. We summarized the patient's information including tumor staging, p16 status, alcohol and tobacco history in Table 2.

**TABLE 2:** Clinical characteristics of patient cohort.

Cohort Demographics & Clinical Characteristics	*n* (%)
**Age (years)**	
Median (range)	61 (36‐80)
Mean	60.7 ± 9.8
**Sex**	
Male	59 (94)
Female	4 (6)
**Primary Tumor Type**	
Squamous Cell Carcinoma	59 (94)
Other	4 (6)
**Primary Tumor location**	
Nasopharynx	5 (8)
Oropharynx	43 (68)
Hypopharynx	3 (5)
Larynx	4 (6)
Unspecified	8 (13)
**p16 status**	
p16+	36 (57)
p16‐	1 (2)
Unknown	26 (41)
**Tumor (T) and Node (N) Staging**	
T_1_	4 (6)
T_2_	22 (35)
T_3_	5 (8)
T_4_	13 (21)
Unspecified N_1_ N_2_ N_3_ Unspecified	19 (30) 8 (13) 28 (44) 4 (6) 23 (37)
**Chemotherapy Regimen**	
Cisplatin	43 (68)
Carboplatin	6 (10)
No chemotherapy	9 (14)
Combination Cis/Carboplatin	5 (8)
**Smoking Habits**	
Smoker	36 (57)
Non‐Smoker	21 (33)
Unspecified	6 (10)
**Drinking Habits**	
Occasional Drinker	23 (36)
Heavy Drinker	17 (27)
Non‐Drinker	13 (21)
Unspecified	10 (16)
**Posttreatment Assessment**	
Complete Responder (CR)	23 (36.5)
Partial Responder (PR)	40 (63.5)

^#^ No statistically significant clinical features found.

### 4.2 Classification performance

We applied AGAMVFS on the training set and ranked features, and sequentially added features by considering forward sequential addition strategy. In this strategy, features were added one by one according to their ranking, and model performance was evaluated at each step. The performances of KNN and SVM are shown in Tables 3 and 4, respectively. We reported KNN performance sequentially such that the first row of Table 3 shows the performance of first top feature, the second row shows top‐2 features and the last row shows the top‐10 features. We obtained the best performance for the KNN classifier using top‐7 features with accuracy = 75% (Specificity = 79%, Sensitivity = 64%, F1‐score = 64%, Balanced‐Accuracy = 72%). Likewise, we obtained the best performance for the SVM classifier using top‐6 features with accuracy = 85% (Specificity = 91%, Sensitivity = 76%, F1‐score = 80%, and Balanced‐Accuracy = 83%).

**TABLE 3:** The performance of the KNN classifier after ranking features using AGAMVFS. Features are sorted in descending order using AGAMVFS, and KNN is applied to train a model to classify CR and PR. Features are sequentially added to classifier.

# Top‐Features	Specificity (%)	Sensitivity (%)	Accuracy (%)	F1‐score (%)	B‐ACC^†^ (%)
1	68	60	66	55	64
2	70	62	68	57	66
3	71	63	69	58	67
4	74	62	70	59	68
5	76	63	72	61	70
6	78	62	74	63	70
7	**79**	**64**	**75**	**64**	**72**
8	77	63	73	63	69
9	79	50	69	53	64
10	79	50	69	53	64

^†^ B‐ACC: Balanced Accuracy.

**TABLE 4:** The performance of SVM classifier after ranking features using AGAMVFS. Features are sorted in descending order using AGAMVFS, and SVM applied to train a model to classify CR and PR. Features are sequentially added to classifier.

# Top‐Features	Specificity(%)	Sensitivity (%)	Accuracy (%)	F1‐score (%)	B‐ACC^†^ (%)
1	84	30	66	37	57
2	89	25	67	34	57
3	89	35	71	45	62
4	89	30	69	40	60
5	92	70	84	76	81
6	**91**	**76**	**85**	**80**	**83**
7	92	65	83	72	79
8	92	60	81	69	76
9	92	60	81	69	76
10	95	60	83	71	77

^†^ B‐ACC: Balanced Accuracy.

### 4.3 Selected features

The top 10 most frequent features were MRI‐GLCM‐Difference‐Average, QUS‐GLRLM‐Gray‐Level‐Nonuniformity‐Normalized, MRI‐GLSZM‐Large‐Area‐Emphasize, QUS‐GLSZM‐Zone‐Entropy, MRI‐GLRLM‐Short‐Run‐Low‐gray‐Level‐Emphasis, CT‐GLCM‐Correlation, QUS‐GLDM‐Dependence‐Variance, QUS‐GLRLM‐Low‐Gray‐Level‐Run‐Emphasis, QUS‐GLCM‐Sum‐Square, QUS‐GLCM‐Difference‐Average. We applied two‐sample t‐test on selected features and showed results in Table 5. Among the top‐10 features, MRI‐GLCM‐Difference‐Average and MRI‐GLSZM‐Large‐Area‐Emphasize were statistically significant.

**TABLE 5:** The results of two‐sample *t*‐test on top 10 features.

Selected Features	p‐value
MRI‐GLCM‐Difference‐Average	**0.001**
QUS‐GLRLM‐Gray‐Level‐Nonuniformity‐Normalized	0.56
MRI‐GLSZM‐Large‐Area‐Emphasize	**0.01**
QUS‐GLSZM‐Zone‐Entropy	0.09
MRI‐GLRLM‐Short‐Run‐Low‐gray‐Level‐Emphasis	0.07
CT‐GLCM‐Correlation	0.25
QUS‐GLDM‐Dependence‐Variance	0.72
QUS‐GLRLM‐Low‐Gray‐Level‐Run‐Emphasis	0.20
QUS‐GLCM‐Sum‐Square	0.31
QUS‐GLCM‐Difference‐Average	0.48

**FIGURE 3:**
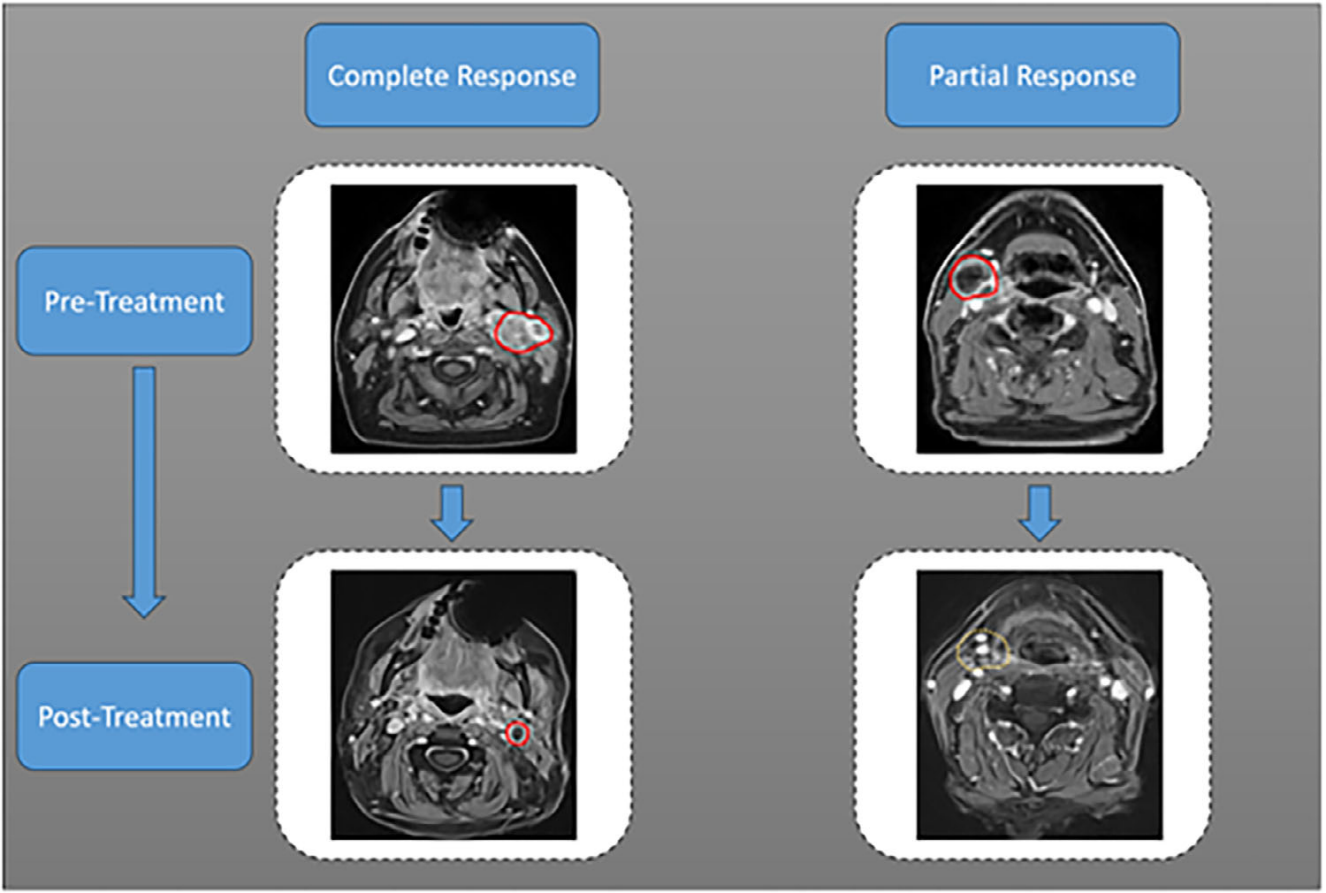
Pretreatment (before radiation therapy) and posttreatment (after radiation therapy) of axial T1‐weighted MRI images for a patient with complete response (CR) and a patient with partial response (PR).

**FIGURE 4:**
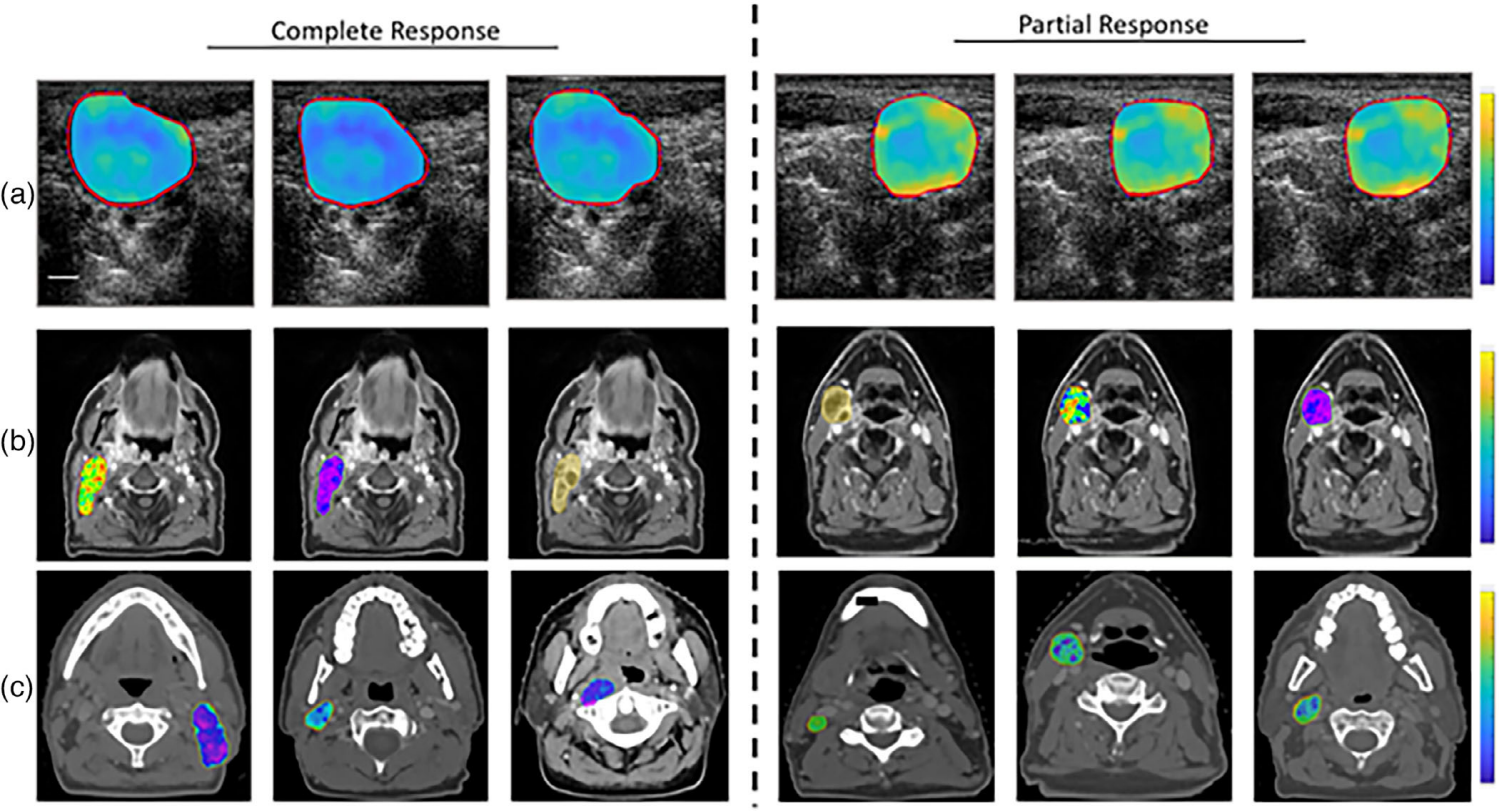
Parametric maps for the two response groups: (a) Mid‐Band Fit parametric maps (range from ‐10 to 40 dB), (b) MRI‐GLCM‐Difference‐Average (with range [0‐4]), and (c) CT‐GLCM‐Correlation (with range [0‐1]). Scale bar is 5 mm.

Based on Table 5, the top feature is MRI‐GLCM‐Difference‐Average, which is statistically significant. In this regard, Figure 3 represents the MRI scans with the region of interest before and after RT treatment for two patients with CR and PR responses. Additionally, Figure 4 shows the parametric maps of QUS, MRI, and CT for the two response groups (PR and CR).

### 4.4 Comparison with single image modality

We compared our proposed method with a single imaging modality to show the effectiveness of a multi‐modality approach in predicting response to treatment for patients with H&N cancers. Table 6 shows the performance of the proposed method and a single imaging modality. For a single imaging modality, we applied mRMR feature selection followed by sequential feature selection (SFS) using an SVM classifier. Based on Table 6, Multi‐modality (AGAMVFS), which is the SVM with top‐6 features, outperforms QUS, CT, and MRI modalities. The best accuracy was obtained by multi‐modality (accuracy = 85). QUS, MRI, and CT obtained accuracy = 79, accuracy = 79 and accuracy = 69, respectively. The values of “Multi‐modality: AGAMVFS” in Table 6 are results of the SVM top‐6 using AGAMVFS which can be found in Table 4.

**TABLE 6:** The comparison between multi‐modal imaging and single‐modal imaging for prediction of response to treatment for patients with H&N cancers.

Modality \ metrics	Sensitivity (%)	Specificity (%)	Accuracy (%)	B‐ACC (%)
QUS	81	75	79	78
CT	70	68	69	69
MRI	85	70	79	77
Multi‐modality: AGAMVFS	**91**	**76**	**85**	**83**

B‐ACC: Balanced Accuracy.

### 4.5 Comparison of AGAMVFS with state‐of‐the‐art feature selection techniques

We compared AGAMVFS technique with different approaches and seven state‐of‐art feature selection techniques. We listed techniques and approaches as follows:

1. mRMR‐SFS: All features are concatenated and the mRMR followed by SFS is applied.
2. NMFFS^39^: Nonnegative matrix feature selection (NMFFS) is an unsupervised feature selection. In this technique, matrix of features is decomposed to feature weight matrix and representation matrix. The feature weight matrix with orthogonality constraint is an indicator matrix which can be used to feature selection.
3. AUEFS^58^: Autoencoder inspired unsupervised feature selection (AUEFS). AUEFS has on layer encoder and one layer decoder. The selected features are obtained by applying group‐LASSO row sparsity regularization on weight of encoding phase.
4. UFGOR^59^: Unsupervised Feature Selection Guided by Orthogonal Representation (UFGOR). UFGOR is a technique based on NMF which is applied to orthogonalized features. In this technique, matrix of features is orthogonalized by gram‐schmidt orthogonalization and then NMF is applied for feature selection.
5. OCMFLP^60^: Orthogonally constrained matrix factorization with local preserving (OCMFLP) is an unsupervised feature selection. In this technique, data is projected to parameter matrix and basis matrix such that both are constrained to be orthogonal. To ameliorate the outlier and noise effects, $l_{2,1}$ norm is applied for error reconstruction. Fixed Laplacian graph and $l_{2,1}$ sparse regularization are employed.
6. CCSFS^61^: Consensus cluster structure guided multiview unsupervised feature selection (CCSFS) is a MVFS technique. CCSFS generates cluster structures based on the number of views and makes a consensus structure by fusing all generated cluster structures. A projection matrix is defined for each view, which is called feature weight matrix, to map data from each view to the consensus cluster structure by making a regression model. Features can be ranked by applying group‐LASSO row sparsity regularization on feature weight matrix.
7. PLMWSC^62^: Partition level multiview subspace clustering (PLMWSC) is an unsupervised mutli‐view clustering technique. In this technique, the affinity graph matrix is learned by self representation property for each view separately. In next step, the spectral clustering is employed to obtain final clustering. In the last step, fusion partition is applied to the consensus cluster indicator matrix and cluster indicator matrix to minimize the squared distance between a partition and the consensus clustering matrix.

We used SVM as classifier for all features and showed results of prediction (PR and CR) in Table 7. The best performance was reported for each method.

**TABLE 7:** The results of AGAMVFS and comparative techniques to predict CR and PR for patients’ with H&N.

Technique \ Metric	Sensitivity (%)	Specificity (%)	Accuracy (%)	B‐ACC (%)
mRMR‐SFS	81	75	79	78
NMFFS	80	71	75	74
AUEFS	83	75	80	78
UFGOR	82	73	77	76
OCMFLP	85	75	81	79
CCSFS	87	74	82	81
PLMWSC	86	73	81	80
AGAMVFS	**91**	**76**	**85**	**83**

Based on Table 7, AGAMVFS, as a multiview feature selection technique, outperforms all comparative techniques (Sensitivity = 91%, Specificity = 76%, Accuracy = 85%, and B‐ACC = 83%). Additionally, CCSFS obtained the second‐best performance (Sensitivity = 87%, Specificity = 74%, Accuracy = 82%, and B‐ACC = 81%), which shows the effectiveness of multiview feature selection techniques. The NMFFS ranked last in terms of performance (Sensitivity = 80%, Specificity = 71%, Accuracy = 75%, and B‐ACC = 74%). OCMFLP was the best single view feature selection technique (Sensitivity = 85%, Specificity = 75%, Accuracy = 81%, and B‐ACC = 79%). However, AGAMVFS had 4% higher balanced accuracy than OCMFLP.

### 4.6 The analysis of parameter $\mu_{v}$

The parameter $\mu_{v}$, which is a weight factor to distinguish different views, shows the contribution of each modality in feature selection and it is updated by (22). At the end of the process, after convergence, the final $\mu_{v}$ shows the weight of each modality. In this study, for QUS, CT and MRI modalities, we obtained $\mu_{QUS}=0.9$, $\mu_{MRI}=0.073$ and $\mu_{CT}=0.027$. Additionally, in the top 10 features, which are shown in Table 5, QUS, MRI, and CT had six features, three features, and one feature, respectively.

## 5 DISCUSSION

In this study, we proposed a new multiview feature selection technique to predict the response to RT treatment for patients with H&N cancers using QUS, MRI, and CT radiomic features. We determined 350 QUS radiomic features, 70 CT radiomic features, and 70 MRI radiomic features from 63 patients, which indicate that we have high‐dimensional dataset such that the number of features is significantly greater than the number of samples. Although feature extraction techniques such PCA and LDA can be applied to reduce dimensionality, the interpretability would be lost using feature extraction which is very important in clinical studies. Additionally, for multi‐modal imaging, conventional feature selection techniques such as mRMR concatenate features of all modalities naively, therefore they fail to obtain the most informative features due to losing correlation among modalities. Nevertheless, we proposed AGAMVFS technique, which is multiview feature selection technique, to select the most discriminative features among all three modalities (QUS, MRI, and CT) to improve the learning model to distinguish patients with CR and patients with PR.

### 5.1 Multi‐modal imaging versus single‐modal imaging

We compared multi‐modal imaging with single‐modal imaging and results proved that the accuracy of prediction was improved by multi‐modal imaging compared to single‐modal imaging. The accuracy of multi‐modal imaging was 6% higher than QUS and MRI, and 16% higher than CT. QUS obtained the best result among all imaging modalities and $\mu_{QUS}=0.9$ shows the importance of QUS in predicting of treatment outcomes for patients with H&N cancer. Six out of the top 10 features were derived from QUS, highlighting the significance of QUS radiomic features.

Chinnery et al.^63^ trained a machine learning model using radiomic, clinical, and dosimetric features to predict the need for a replan prior to treatment. They simply concatenated all features and applied random forest for feature selection, which is a supervised embedding‐based feature selection. Although the combination of radiomic, clinical, and dosimetric features improved the performance of the machine learning model, the source of clinical features is totally different from radiomic features (image source) and naive concatenation leads to loss of inter‐modality relationships. Random forest feature selection only ranks the features based on their entropy (information gain or Gini index) and it does not consider correlation and nonlinear interactions among features.

Xiong et al.^64^ fused CT radiomics and clinical features to predict bladder cancer staging. Although the performance of the predictive model was increased by feature fusion, interpretability of the model was lost. Interpretability can be preserved through a feature selection approach, which plays a crucial role in determining the significance of biomarkers for prognostic and diagnostic purposes.

### 5.2 AGAMVFS versus state‐of‐the‐art feature selection technique

Additionally, we compared AGAMVFS with five state‐of‐the‐art single‐view feature selection techniques and one MVFS technique. Based on Table 7, AGAMVFS outperformed mRMR‐SFS, NMFFS, AUEFS, UFGOR, OCMFLP, and CCSFS. A subtle but important point is thatAUEFS is the single view of AGAMVFS without structure learning regularization. Although AGAMVFS and AUEFS employed autoencoder for feature selection, AGAMVFS outperformed AUEFS since it is the multiview approach and considers correlation among modalities, and AGAMVFS utilized Laplacian graph matrix to preserve the geometrical information of samples. Additionally, we considered adaptive graph learning for AGAMVFS, where the affinity matrix of the data is updated in each iteration, and consequently, the Laplacian graph is also updated.

Furthermore, CCSFS and PLMWSC obtained the second‐best performance and the third‐best, respectively, which show the superiority of MVFS over single view feature selection.

Base on Table 7, the NMFFS technique obtained the last rank due to its weakness in using a feature weight matrix as an indicator matrix. NMFFS only considers orthogonality constraint to have feature weight matrix as an indicator matrix, but Saberi et al^65^ showed with example $W^{T}=\left[\begin{array}{ccc} \frac{1}{\sqrt{2}} & 0 & \frac{1}{\sqrt{2}}\\ 0 & 1 & 0 \end{array}\right]$ that orthogonality constraint lonely is not sufficient to have indicator matrix.

mRMR‐SFS is a conventional technique that first ranks the features and then features are sequentially selected. However, ranking features in the original space without considering the combination of features in latent space is not an efficient approach. For instance, if we have three features $x_{1}$, $x_{2}$, and $x_{3}$ and the features are ranked using mRMR based on importance as $x_{1}$ >$\;x_{2}$> $x_{3}$. If we want to take two features to train a model, we will take $x_{1}$ and $x_{2}$. However, Nie et al.^66^ showed that the combination of different features with less importance can provide better discrimination in latent space, which means that the combination of $x_{1}$ and $x_{3}$ can provide better discrimination. mRMR does not consider complex relationships or interactions between multiple features and only works based on pairwise interactions between features and the target variable. Additionally, it is not efficient for high‐dimensional data with non‐linear relationships between features and the target variable since it considers linear relationships in the data.

In UFGOR, there is no structure learning and then the geometrical information of samples cannot be preserved after projection. Additionally, UFGOR is a single‐view features selection, and it does not extract correlation among modalities which plays significant role to obtain the most informative features.

Although OCMFLP employed the $l_{2,1}$ norm instead of the Frobenius norm to handle the effect of noisy outliers, it was still considerably affected by noise and outliers due to the fixed graph learning. In OCMFLP, the affinity matrix is constructed based on the original data, and thus noise and outliers can impact the algorithm.

Although CCSFS is MVFS and obtained the second‐best performance, it cannot preserve the geometrical information of data in embedding space which affects the performance of algorithm.

In terms of multi‐modal features, Welch et al.^67^ combined CT radiomics features and clinical features to train a machine learning model to predict RT outcomes for patients with H&N cancer. However, they naively concatenated CT radiomics features and clinical features without considering the correlation between two the different types of features, which directly impacts the performance of the classifier. In terms of imaging modality, they used only CT radiomics features. Devakumar et al.^68^ combined radiomics features of CT and PET to predict local failure after radiotherapy for patients with locally advanced H&N cancers. Similarly, they naïvely combined features without considering the correlation between two imaging modalities and then applied LASSO regression to select top features. Toe et al.^69^ applied machine learning and ensemble feature selection on clinicopathologic and dosimetric data to identify the risk of H&N cancer treatment‐related lymphedema. They applied a single view ensemble feature selection technique to identify features associated with lymphedema incidence. In contrast, the AGAMVFS is a multiview feature selection technique that can keep the correlation between different modalities in the process of feature selection.

In contrast to deep learning, multi‐modality feature selection offers interpretability that aids oncologists in assessing tumor response and thereby adjusting treatment strategies. Deep learning methods, on the other hand, extract features through convolutional filters, lacking interpretability. Even Gradient‐weighted Class Activation Mapping^70^ fails to explain the biological effects of tumors or their correlation with treatment; instead, it highlights significant image areas crucial for classification tasks. Additionally, contribution of each modality cannot be measured using deep learning fusion techniques. whereas parameter μ had a significant role to interpret the contribution of each modality.

QUS‐radiomics had a significant contribution in the prediction of treatment outcome in comparison with MRI and particularly CT. Therefore, we can conclude that features from lymph nodes in H&N cancer (since QUS only extracts LN information) provide considerable information for a machine learning model to learn the pattern of the data robustly and subsequently predict treatment outcome with minimal false prediction. Based on Table 6, QUS obtained the best performance in comparison with CT and MRI, which shows the effectiveness of QUS and lymph node information in predicting of RT treatment outcome for patients with H&N cancer. Adding different types of features like dosimetric^63^ and clinical^69^ can provide more valuable information for the training model to improve the performance of the classifier. Clinical characteristics such as HPV status can be utilized in combination with imaging‐based radiomics to account for the tumor biology of H&N subsites, such as the oropharynx.

A small sample size and data from only one center are two limitations of this study. The generalizability of our classification results can be challenged due to the small sample size. Moreover, we trained our model using data from only one center.

## 6 CONCLUSION

In conclusion, multi‐modal imaging with multiview feature selection can enhance classifier performance in predicting the response to RT for patients with H&N cancer. In this study, we proposed a novel MVFS. AGAMVFS utilizes an autoencoder to learn features across all views and dynamic collaborative graphs to learn all views features and view‐specific features. This technique promotes consistency across various views and results in a shared projection used for feature selection. AGAMVFS was applied to select features among QUS, CT, and MRI radiomic to discriminate between PR and CR outcomes. Moreover, integrating radiomics features from diverse imaging modalities such as MRI, CT, and QUS improves the machine learning classifier's ability to discern data patterns and predict treatment outcomes in patients with H&N cancer. QUS had the highest weight factor μ, indicating its importance in building a predictive model for treatment outcome prediction in patients with head and neck cancer. To the best of our knowledge, this is the first study to use multiview feature selection for outcome predictions in patients with H&N cancers. For future study, clinical information like HPV status can be utilized in combination with imaging‐based radiomic to consider tumor biology of H&N subsites, such as the oropharyngeal.

## CONFLICT OF INTEREST STATEMENT

The authors declare no conflicts of interest.

## Data Availability

Contact to corresponding author.

## APPENDIX A

### A.1

To solve optimization problem (10), we have Lagrangian function of (10) as follows:

(A1)
$$L\;\left(s_{i,:},\;\phi ,\psi \right)=\frac{1}{2}{∥s_{i,:}+\frac{z_{i,:}}{2\eta }∥}_{2}^{2}\;-\phi \left(s_{i,:}^{T}1-1\right)-s_{i,:}\psi$$

where $\psi \in R^{n}\geq 0$ and ϕ∈R are the Lagrangian multipliers. Partial derivative with respect to $s_{i,:}$ is computed as follows:

(A2)
$$s_{ij}+\frac{z_{i,j}}{2\eta }-\phi_{i}^{*}-\;\psi_{ij}^{*}=0$$

where $\phi_{i}^{*}$ and $\psi_{ij}^{*}$ are the optimal values of ϕ and ψ, and we have $s_{ij}\psi_{ij}^{*}=0$ by applying Karush‐Kuhn‐Tucker (KKT) conditions.^51^ Therefore, the solution of $z_{i,j}$ is obtained as follows:

(A3)
$$\;s_{ij}=T\left(-\frac{z_{i,j}}{2\eta }+\phi_{i}^{*}\right)$$

where function T is defined as follows:

(A4)
$$T\;\left(x\right)=\left\{\begin{array}{c} x,\;if\;x\geq 0\\ 0,\;if\;x<0 \end{array}\right.\;$$

Additionally, since $s_{i,j}\geq 0$, $\sum_{j=1}^{n}s_{i,j}=1$ and only k elements are updated, we can obtain $\phi_{i}^{*}$ as follows:

(A5)
$$\begin{array}{cc} \;\sum_{j=1}^{k}s_{i,j} & =\sum_{j=1}^{k}\left(\phi_{i}^{*}-\frac{z_{i,j}}{2\eta }\right)\\ & =k\phi_{i}^{*}-\;\sum_{j=1}^{k}\frac{z_{i,j}}{2\eta }=1\Rightarrow \;\phi_{i}^{*}=\frac{1}{k}+\frac{1}{2\eta k}\sum_{j=1}^{k}z_{i,j} \end{array}$$

$\sum_{j=1}^{k}s_{i,j}$ represents *j* elements of *i*‐th row of matrix S.

Based on (A4) and (A5), $s_{i,k+1}=0$ (due to constraint $∥s_{i,:}{∥}_{0}=k$) and $s_{i,:}$ has exact k‐nonzero elements if and only f $\eta_{i}=\frac{k}{2}\;s_{i,k+1}-\frac{1}{2}\sum_{j=1}^{k}s_{ij}$. Consequently, by having $\phi_{i}^{*}$ and η, the solution of $s_{ij}$ is derived as follows:

(A6)
$$\;s_{ij}=T\left(\frac{z_{i,k+1}-z_{ij}}{kz_{i,k+1}-\sum_{j=1}^{k}z_{ij}}\right)$$

As a result, $s_{ij}=0$ for j≥k+1 since $z_{ij}>z_{i,k+1}$ and $\frac{z_{i,k+1}-z_{ij}}{kz_{i,k+1}-\sum_{j=1}^{k}z_{ij}}<0$. This can be interpreted that a sample is disconnected with the n−k samples with largest values of $z_{ij}$ and only connected to k neighbors with the similarity of $s_{ij}=T\left(\frac{z_{i,k+1}-z_{ij}}{kz_{i,k+1}-\sum_{j=1}^{k}z_{ij}}\right)$.

Additionally, is updated in each iteration as following:

(A7)
$$\eta =\sum_{i=1}^{n}\eta_{i}\;$$

where n is the number of samples.

## APPENDIX B

### B.1

To solve optimization problem (13), the Lagrangian function can be expressed as follows:

(B1)
$$L\;\left(\mu ,\;\tau ,\rho \right)=\sum_{v=1}^{3}\mu_{v}^{\gamma }E^{\left(v\right)}\;-\tau \left(\mu^{T}1-1\right)-\rho^{T}\mu$$

where $\tau \in R^{n}\geq 0$ and ρ∈R are Lagrangian multipliers. By taking partial derivative with respect to μ and set it equal to zero $\frac{\partial L}{\partial \mu }=0$, we have

(B2)
$$\gamma \mu_{v}^{\gamma -1}E^{\left(v\right)}-\tau^{*}-\;\rho^{*}=0$$

where $\tau^{*}$ and $\rho^{*}$ are the optimal τ and ρ, respectively. By applying KKT condition, the solution of μ for each view is obtained as follows:

(B3)
$$\;\mu_{v}={\left(\frac{\tau^{*}+\rho^{*}}{\gamma E^{\left(v\right)}}\right)}^{\frac{1}{\gamma -1}}\;$$

Based on $\mu_{v}\geq 0$ and $\mu^{T}1=\sum_{v}\mu_{v}=1$ constraints, and $\rho^{*}\;\mu_{v}=0$ (KKT condition), we have

(B4)
$$\;\sum_{v}{\left(\frac{\tau^{*}}{\gamma E^{\left(v\right)}}\right)}^{\frac{1}{\gamma -1}}=1\Rightarrow \;\tau^{*}=\frac{1}{\sum_{v=1}^{3}{\left(E^{\left(v\right)}\right)}^{\frac{1}{1-\gamma }}}\;$$

Therefore, we obtain

(B5)
$$\;\mu_{v}=\frac{{\left(E^{\left(v\right)}\right)}^{\frac{1}{1-\gamma }}}{\sum_{v=1}^{3}{\left(E^{\left(v\right)}\right)}^{\frac{1}{1-\gamma }}}\;$$

In each view, we have $\mu_{v}\geq 0$ since $s_{ij}$> 0 and $∥U^{{\left(v\right)}^{T}}x_{i}^{\left(v\right)}-U^{{\left(v\right)}^{T}}x_{j}^{\left(v\right)}{∥}_{2}^{2}$ is positive ($E^{\left(v\right)}\geq 0)$. Consequently, the constraint $\mu_{v}\geq 0$ is satisfied.
